# Establishment of a Dihydrofolate Reductase Gene Knock-In Zebrafish Strain to Aid Preliminary Analysis of Congenital Heart Disease Mechanisms

**DOI:** 10.3389/fcvm.2021.763851

**Published:** 2021-12-15

**Authors:** Ke Gong, Ting Xie, Yifeng Yang, Yong Luo, Yun Deng, Kun Chen, Zhiping Tan, Hui Guo, Li Xie

**Affiliations:** ^1^Department of Cardiovascular Surgery, The Second Xiangya Hospital of Central South University, Central South University, Changsha, China; ^2^State Key Laboratory of Developmental Biology of Freshwater Fish, Hunan Normal University, Changsha, China; ^3^College of Life Sciences, Hunan Normal University, Changsha, China; ^4^The Clinical Center for Gene Diagnosis and Therapy of The State Key Laboratory of Medical Genetics, The Second Xiangya Hospital of Central South University, Central South University, Changsha, China

**Keywords:** zebrafish, CRISPR/Cas9, heart development, congenital heart disease, *DHFR* gene

## Abstract

**Background:** The dihydrofolate reductase (*DHFR*) gene is imperative in development, therefore it is essential to explore its effects on heart development. Thus, here a *dhfr* zebrafish knock-in (KI) strain was constructed.

**Methods:** CRISPR/Cas9 technology was used to establish the *dhfr* KI zebrafish strain. This strain was hybridized with TgG fluorescent strain zebrafish to observe the phenotypes of heart shape, size, and circularization direction. Wild-type (WT) and KI zebrafish were then dissected and histologically stained to observe pathological changes. Western blot analysis was used to verify the increased expressions of zebrafish genes after KI. Hybridization experiments were used to confirm the presence of abnormal gonadal dysplasia.

**Results:** The zebrafish *dhfr* KI strain was successfully constructed through CRISPR/Cas9 technology. At 6 days post fertilization (dpf), microscopic examinations of KI (homozygous) specimens revealed pericardial effusions, heart compressions, and curled tails. Compared with WT, the Hematoxylin and Eosin (H&E) tissue sections of KI-homozygous zebrafish showed defects such as reduced atria and ventricles. Western blot analysis indicated that the expression of the DHFR protein increased in both heterozygotes and homozygotes of *dhfr* KI zebrafish. Hybridization experiments revealed that *dhfr* KI may affect gonadal function.

**Conclusion:** The *DHFR* gene plays an important regulatory role in the process of heart development, and copy number variations (CNVs) of this gene may constitute a new pathogenic mechanism of congenital heart disease (CHD).

## Introduction

Congenital heart disease (CHD) is one of the most common congenital malformations in fetuses. Its development process is regulated by the expressions of a variety of genes. The abnormal expressions of these genes can lead to different types of heart defects (1). Pulmonary Atresia (PA) is an extreme type of right ventricular system CHD; it is a kind of conical malformation. The anatomical lesions of PA can affect almost all right heart structures, including the atrioventricular valve, ventricular outflow tract, pulmonary valve, and pulmonary artery. The natural prognosis for children with PA is extremely poor (2). Most patients require multiple operations or interventions, and their long-term prognoses may involve developmental delays, developmental delay, and an overall unsatisfactory quality of life and life span. This can become a severe burden on the patient's family, and on society (3, 4). Identifying the regulatory genes in heart development will help to better understand the pathogenesis of PA and aid investigations into its treatment options.

With the continuous advancements in genetic testing and editing technologies, more and more disease-causing genes and their mechanisms are becoming familiar to researchers. Copy number variations (CNVs) are defined as an important genetic mechanism of human phenotypic heterogeneity; their deoxyribose nucleic acid (DNA) variations range from >1 Kbp to several Mbp (5, 6). Recently, some studies have reported that the mutation of the dihydrofolate reductase (*DHFR*) gene (OMIM#126060) mainly causes megaloblastic anemia caused by dihydrofolate reductase deficiencies (7). At the same time, some studies have shown that gene CNVs may also cause cell and animal resistance to the anti-cancer drug methotrexate (8–10). In addition, studies have shown that *DHFR* affects cell proliferation and apoptosis by regulating gene transcription, and that it plays a key role in the development of the hearts and outflow tracts of zebrafish (*Danio rerio*) (11). A previous study performed array detection on 82 PA patients, revealing that one patient had a *DHFR* gene CNV (12). This *DHFR* gene CNV could potentially play an important role during heart development, and thus contribute in the pathogenicity mechanism of PA. Therefore, the present study used CRISPR/Cas9 gene editing technology to construct a *dhfr* knock-in (KI) model for zebrafish, which are model organisms, to verify this hypothesis and preliminarily explore the pathogenesis of PA.

## Materials and Methods

### Ethics Review

The animal study was reviewed and approved by the Ethics Committee of the Second Xiangya Hospital of Central South University (2021123).

### Target Construction

**Figure 1:**
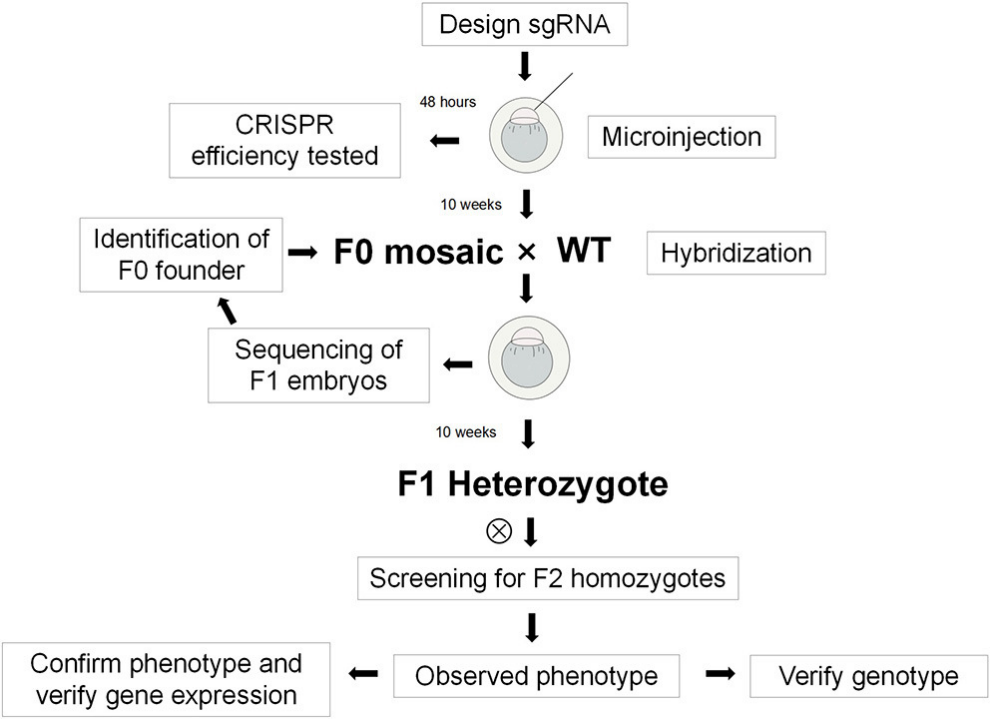
Flow chart of the experiment.

According to the provided species, gene name, or gene identification (ID), the National Center for Biotechnology Information (NCBI; https://www.ncbi.nlm.nih.gov/gene/81882) and Ensemble databases were used to search for related genes, species information, and coding sequence (CDS) gene areas. The corresponding genome structures were analyzed to confirm the exon part of each CDS. According to the nature of each gene, the exon region contained in the statistical gene was identified as the candidate KI site. After analyzing the nature of the gene itself and the encoded protein structure, the exon was placed after the initiation codon ATG and the exon region was placed before the selection. Finally, select sites with low off-target rates were identified as targets for gene KI. The entire process diagram is shown in Figure 1.

### Design of Guide Ribose Nucleic Acid and Identification Primer

**Figure 2:**
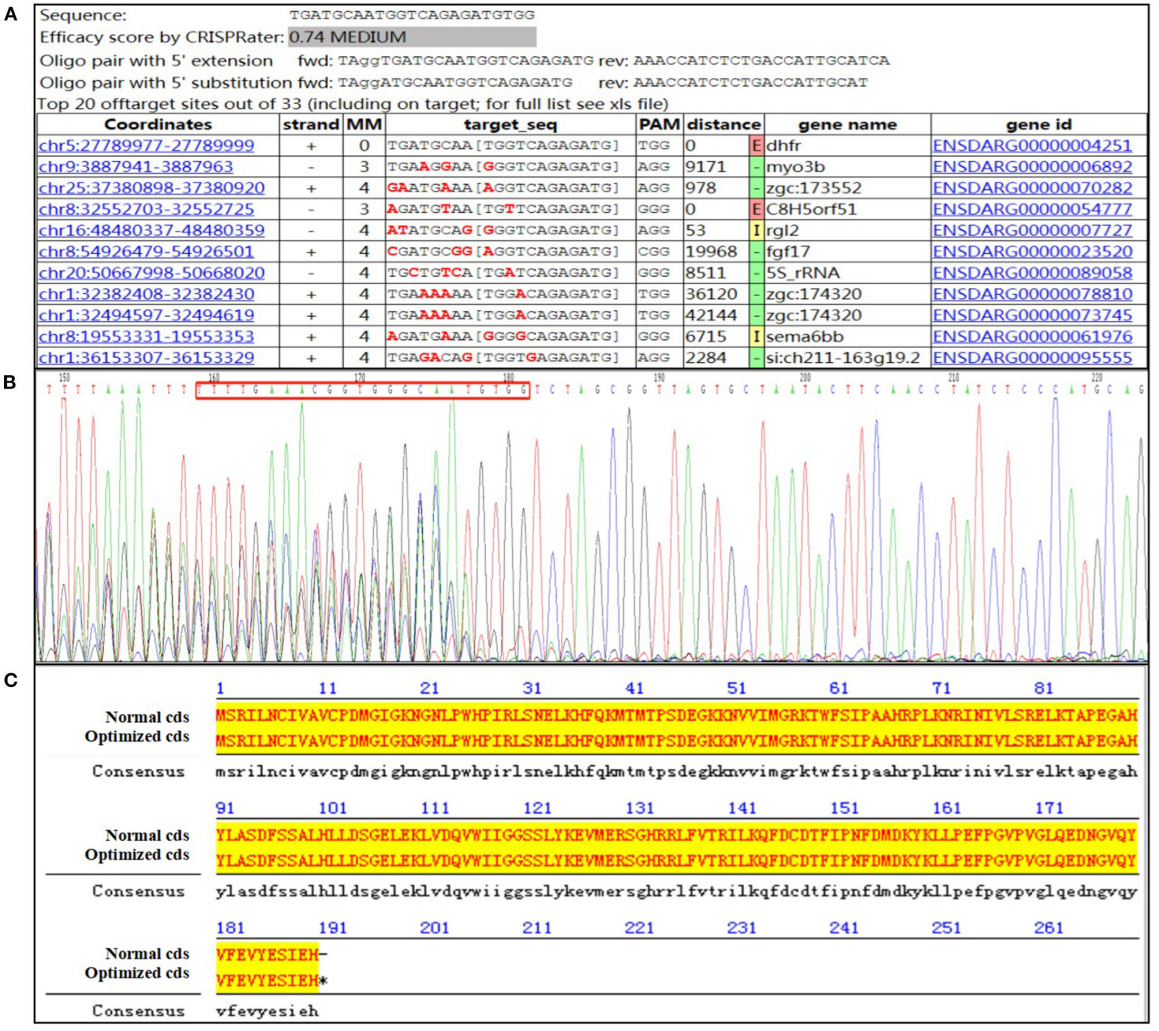
Design of guide RNA and identification primer. **(A)** Guide #5 TGATGCAATGGTCAGAGATGTGG; **(B)** the sequencing result of the activity verification; **(C)** the optimized amino acid sequence alignment (the lowercase font is the optimized base).

According to the protein-conserved functional domain and genetic structure, the target sequence to be knocked-in comprised a small guide RNA (sgRNA) recognition sequence on coding exon 6 that was designed to realize KI. GuideRNA: 5′-TGATGCAATGGTCAGAGATGTGG-3′ (*DHFR*-sgRNA5; Figure 2). The following polymerase Chain Reaction (PCR) primers were designed for the target region: 5′-GGCACCCTATCAGACTGAGC-3′ (*dhfr*-trans-F1) and 5′-CCTGCATTGGACACACCAAAAT-3′ (*dhfr*-3'KI-R1).

### KI Donor Design

The *dhfr* zebrafish genome sequence (*dhfr*-201) was downloaded from the Ensembl online database. The *dhfr* CDS codon was merged and optimized through the Codon Usage Database to avoid the appearance of repetitive sequences (http://www.kazusa.or.jp/codon/cgi-bin/showcodon.cgi?species=7955).

### *In vitro* Transcription of SgRNA and Cas9mRNA

2 × Mastermix (10 μL), ultrapure water (7 μL), forward and reverse primers (F1 and R1) 1 μl (5 μM), and zebrafish genomic DNA (50 ng·μL^−1^) were used to amplify the guide RNA; the amplified product was used for gene sequencing. A T7 *in vitro* transcription kit was used to perform the *in vitro* transcription of sgRNA, using the product as a template. RNA was precipitated with absolute ethanol, following which ultrapure water treated with diethylpyrocarbonate was used for resuspension. The resuspended liquid was stored in a refrigerator at −80°C for later use.

The T7-driven humanized Cas9 coding sequence was used as a template and T7 RNA polymerase was used for Cas9 *in vitro* transcription. It was then capped and tailed. RNA was precipitated with absolute ethanol and resuspended in ultrapure water treated with diethylpyrocarbonate. The resuspension liquid was stored in a refrigerator at −80°C for later use.

### Microinjection

A semi-automatic microinjector was used to inject the mixed liquid of the above-mentioned sgRNA (final concentration of 50 ng·μL^−1^) and Cas9mRNA (final concentration of 250 ng·μL^−1^) into the zebrafish zygotes. At the same time, some wild-type (WT) embryos of the same batch were collected as a control group. The embryos were then placed in an incubator (temperature = 28°C) and incubated for 48 h. When the embryos developed to 24 h post-fertilization (hpf), they were taken to prepare a DNA template. The PCR products were sub-cloned into the pGEM-TEasy vector, via amplification with forward and reverse primers. The single transformed clones were subjected to PCR and then sequenced. Finally, the obtained sequence was compared with the WT sequence provided by the NCBI Basic Local Alignment Search Tool (BLAST) to determine the specific site of the mutation.

### Screening and Identification of Founder Generation Zebrafish

The same batch of embryos that tested positive for the effectiveness of the injection was grown to 3 months of age. Part of the tail fin (1–2 mm) of each Founder generation zebrafish was clipped. Lysate was used to extract genomic DNA. The above-designed positive and negative primers were used for PCR amplification, following which the PCR products were subjected to Sanger sequencing. The single peak identified in the sequencing results showed that the corresponding Founder generation zebrafish had hit the target, confirming that the construction was successful.

### Establishment of F1 Generation Zebrafish Strain

Founder generation zebrafish were crossed with WT zebrafish to obtain the F1 generation. Tail trimming was performed on F1 generation zebrafish to identify their genotypes. The birth of a positive zebrafish indicated that the transgene has been integrated into the germ cells, marking the establishment of a zebrafish line with the *dhfr* KI.

### Identification and Dissection of F2 Generation Zebrafish

The F2 generation was obtained from the heterozygous F1 generation. The embryonic heart development and phenotype were observed at 6 days post-fertilization (dpf), and the F2 generation zebrafish were tail-cut and sequenced. The 6-dpf zebrafish were stained with Hematoxylin and Eosin (H&E), and the morphologies, sizes, and circularization directions of their hearts were observed under a microscope. The identification primers were as follows: forward primer, 5′-AGGACAATGGTGTTCAGTATG-3′ (*DHFR*-F); reverse primer, 5′-GCATGGGAGATAGGTTGAAG-3′ (*DHFR*-R).

### Observation of Phenotypes of the Homozygous *dhfr* of the TgG Fluorescent Strain

F1 generation heterozygous females and WT TgG strains of fluorescent zebrafish males were crossed to obtain the F2 generation. After screening and identifying genotypes *via* fluorescence microscopy, F2 generation fluorescent heterozygous zebrafish were screened out. F2 generation zebrafish underwent heterosexual mating to obtain F3 generation fluorescent zebrafish offspring, the phenotypes of which were observed.

### Western Blot Analysis

A 3-month-old zebrafish was dissected to obtain a heart sample. The heart was washed with cold phosphate-buffered saline (PBS), and then lysed with radioimmunoprecipitation assay (RIPA) lysis buffer (Thermo Fisher Scientific, 89901) containing a mixture of 1% protease and phosphatase inhibitor (Thermo Fisher Scientific, 78442). According to the manufacturer's protocols, the protein concentration of the lysate was measured using a total protein quantification (BCA) kit (Thermo Fisher Scientific, 23227). The protein sample (30 μg) was separated *via* 10% sodium lauryl sulfate-polyacrylamide gel electrophoresis, and then transferred to a polyvinylidene fluoride membrane. The membrane was sealed with 5% skimmed milk for 1.5 h, before being incubated with the specific primary antibodies DHFR (Proteintech, 15194-1-AP) and GAPDH (Proteintech, 10494-1-AP) at 4°C overnight. The next day, after being washed thrice with Tris-Buffered Saline and Tween 20 (TBST), the membrane was incubated with IgG secondary antibodies (Abcam, ab97051) at 37°C for 1 h, and then washed again with TBST thrice. Enhanced chemiluminescence (ECL) reagent (Thermo Fisher Scientific, 34580) was used to detect antibody-binding proteins. ImageJ software was used to quantitatively analyze the gray scale of each band.

### Fertility Evaluation

Adult age-matched WT Tuebingen (TU) strain (*n* = 12) and homozygous zebrafish (*n* = 12) were crossed regularly (five crosses in total) to explore the fertility of homozygotes with *dhfr* KI. The following crosses (three pairs per condition) were performed: WT incross, KI incross, WT female crossed with KI male, and KI female crossed with WT male. The sample size was determined as the maximum number of crossings actually possible. The numbers of eggs per fertilization event were assessed for each genotype. The inclusion criteria for fertility analysis were as follows: a successful cross was defined as an event where each paired mating produced >10 fertilized eggs. Infertility was defined as an event in which there was no fertilized egg after three repetitions with the same result. This standard was established before the start of the experiment.

### Statistical Analyses

Independent *t*-tests (two-tailed) were used to evaluate the statistical significances of the differences between two groups, and one-way analysis of variance (ANOVA) was used to evaluate the statistical significances of the differences between two or more groups. For all tests, *p* < 0.05 were considered to be statistically significant.

## Results

The edited Founder generation zebrafish were crossed with WT zebrafish using CRISPR/Cas9 gene-editing technology, and the Founder generation zebrafish were mated. F1 generation KI zebrafish organisms were identified, confirming that the *dhfr* KI zebrafish model organisms and strains were successfully constructed.

### Screening and Identification of F1 Generation Zebrafish

**Figure 3:**
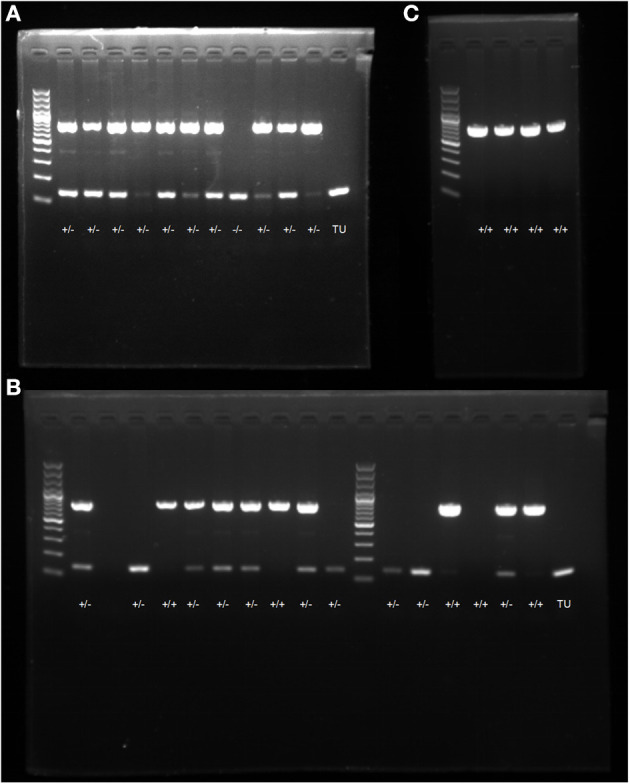
PCR electrophoresis diagram. **(A)** screening and genotype identification of F1 generation zebrafish; **(B)** identification of positive expressions of genotypes in zebrafish; **(C)** self-progeny genotype identification of F1 generation heterozygous zebrafish.

Through the DNA extraction, PCR amplification, electrophoresis, and gene identification of F1 generation zebrafish, WT individuals were found to have a 150 bp band, heterozygotes had two bands at 750 and 150 bp, and homozygotes had one band at 750 bp. Eleven candidate *dhfr* KI F1 generations were identified, 10 of which were confirmed as *dhfr* KI (two females and eight males; Figure 3A).

### Morphological Observations of F2 Generation Zebrafish

**Figure 4:**
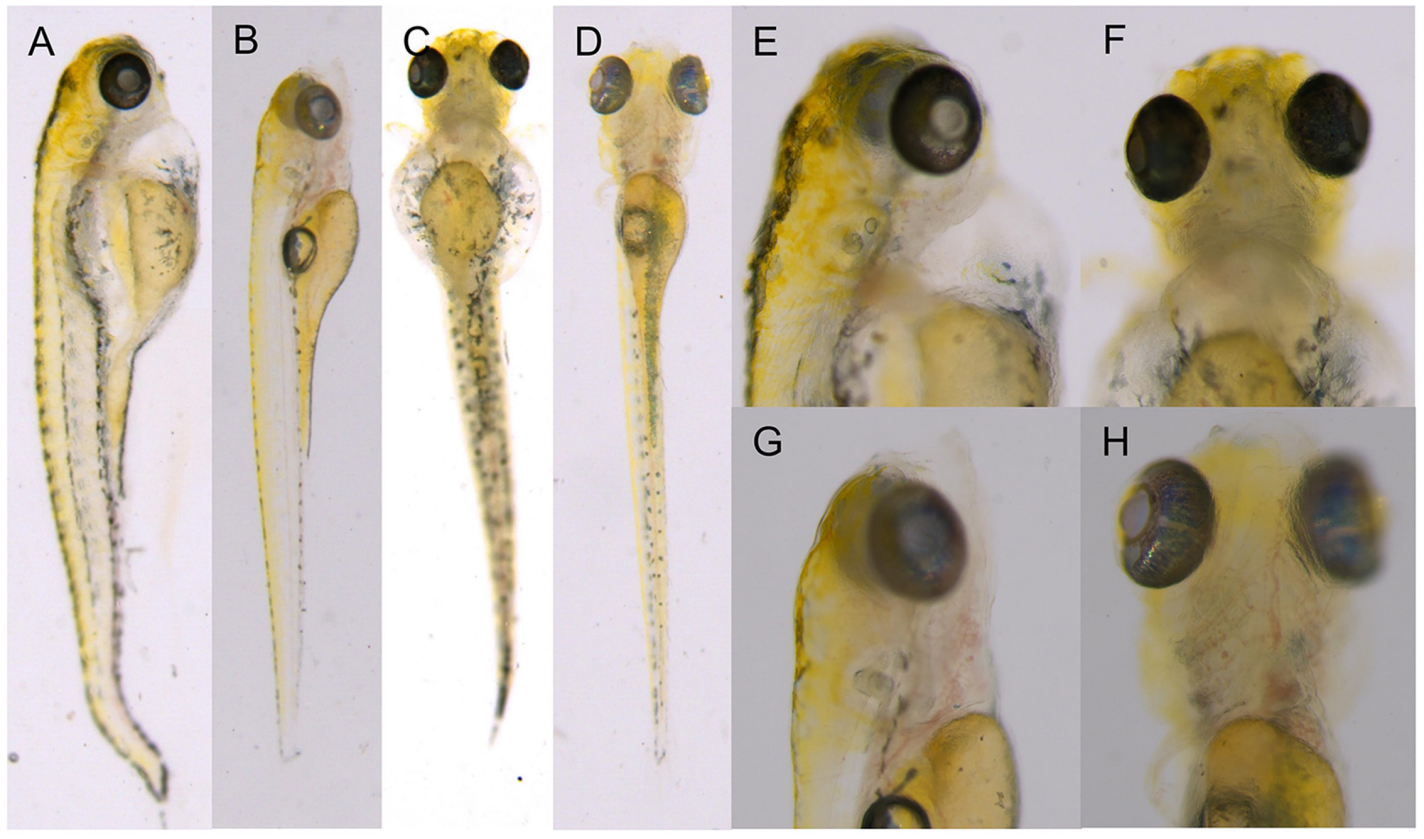
F2 generation zebrafish KI homozygotes at 6 dpf. **(A,C,E,F)** F1 heterozygous self-progeny. **(B,D,G,H)** self-progeny of TU.

F2 generation zebrafish were mated to obtain the F3 generation. The genotype identification results of 16 embryos at 48 hpf showed that the hybridization results were in line with Mendelian inheritance (Figure 3B). At 6 dpf, F2 generation zebrafish KI homozygotes showed abnormal phenotypes such as severe heart compressions, deformations, pericardial edemas, and slow blood flow. Normal-sized atria and ventricles were observed in the WT zebrafish heart areas, as were normal-sized pericardial cavities and blood circulation. However, the transparency around the heart areas of individuals with KI increased significantly, and no normal-sized atria and ventricles were seen. The enlarged peripheral cavity and the heart were deformed under pressure, and the blood circulation in the visual field was abnormal (it decreased or disappeared; Figure 4). The genotyping results showed that all fishes with cardiac abnormalities were homozygous (Figure 3C).

### Gene Sequencing and Identification of F2 Generation Zebrafish

**Figure 5:**
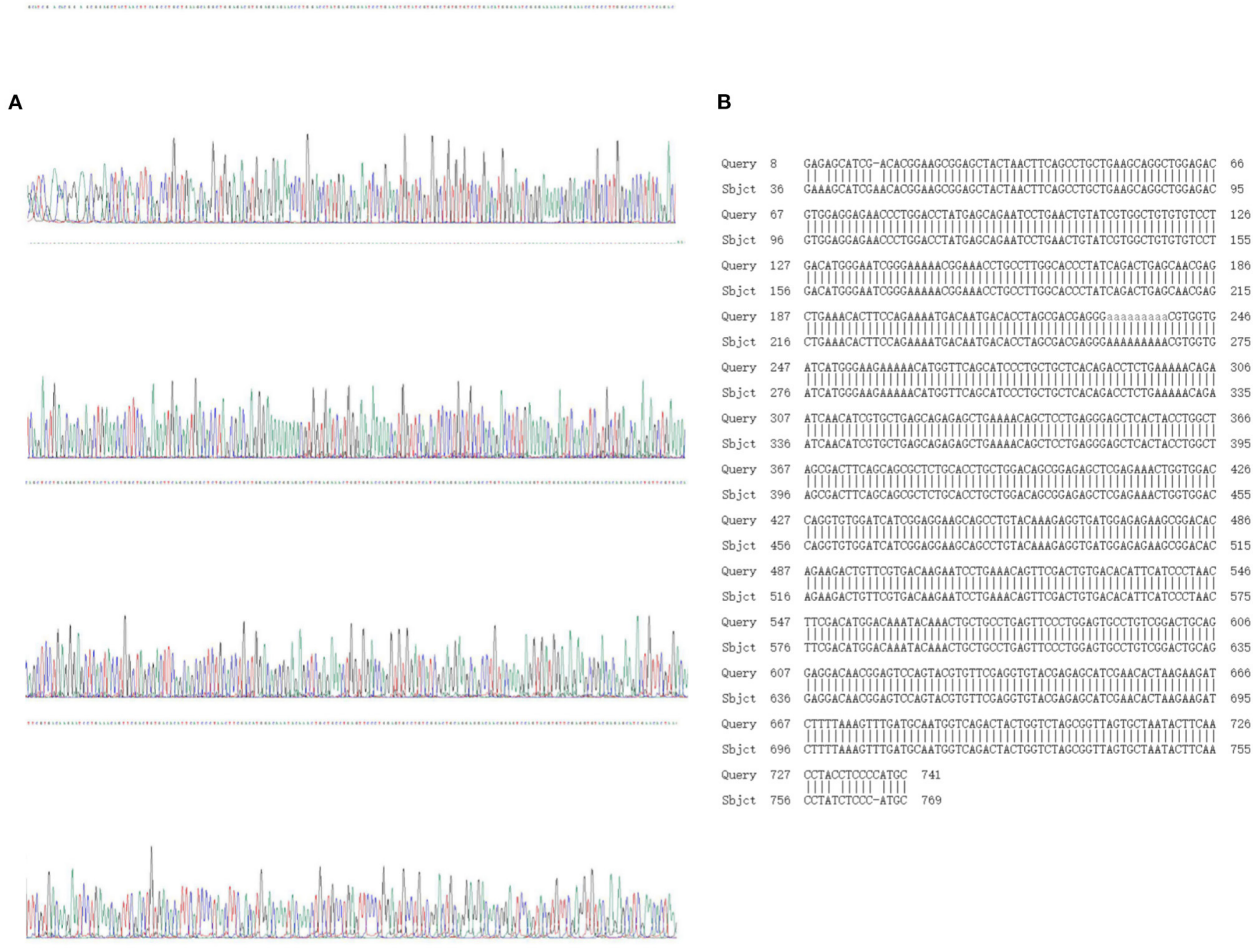
Sanger sequencing and sequence alignment analysis results. **(A)** Sanger sequencing results; **(B)** design sequence and sequencing alignment results.

Sanger sequencing and sequence comparison analysis showed that both WT zebrafish and homozygous KI individuals exhibited a “single peak” waveform, with a 639 bp base sequence knocked in; this is an optimized CDS sequence. The sequence specific KI was as follows: 5′-GGAAGCGGAGCTACTAACTTCAGCCTGCTGAAGCAGGCTGGAGACGTGGAGGAGAACCCTGGACCTATGAGCAGAATCCTGAACTGTATCGTGGCTGTGTGTCCTGACATGGGAATCGGGAAAAACGGAAACCTGCCTTGGCACCCTATCAGACTGAGCAACGAGCTGAAACACTTCCAGAAAATGACAATGACACCTAGCGACGAGGGAAAAAAAAACGTGGTGATCATGGGAAGAAAAACATGGTTCAGCATCCCTGCTGCTCACAGACCTCTGAAAAACAGAATCAACATCGTGCTGAGCAGAGAGCTGAAAACAGCTCCTGAGGGAGCTCACTACCTGGC TAGCGACTTCAGCAGCGCTCTGCACCTGCTGGACAGCGGAGAGCTCGAGAAACTGGTGGACCAGGTGTGGATCATCGGAGGAAGCAGCCTGTACAAAGAGGTGATGGAGAGAAGCGGACACAGAAGACTGTTCGTGACAAGAATCCTGAAACAGTTCGACTGTGACACATTCATCCCTAACTTCGACATGG ACAAATACAAACTGCTGCCTGAGTTCCCTGGAGTGCCTGTCGGACTGCAGGAGGACAACGGAGTCCAGTACGTGTTCGAGGTGTACGAGAGCATCGAACACTAA-3′ (Figure 5).

### Observation of the Phenotype of *dhfr* Homozygous TgG Fluorescent Strain

**Figure 6:**
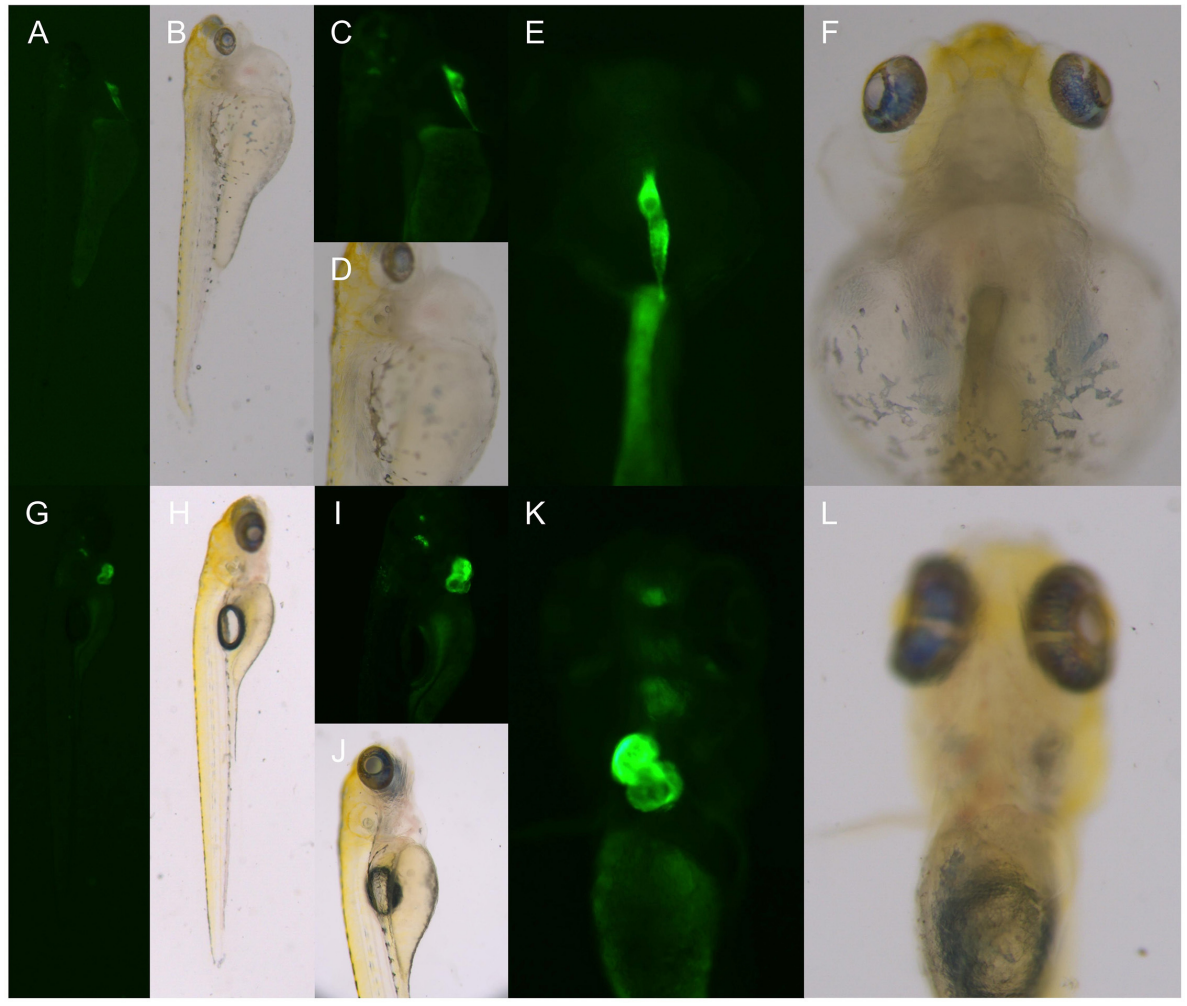
The phenotype of the hybrid offspring of the TgG fluorescent line. **(A–F)** TgG homozygous fluorescent strain *dhfr*. **(G–L)** TgG fluorescent strain TU.

At 6 dpf, the F2 generation zebrafish KI homozygotes of the TgG fluorescent strain showed abnormal phenotypes such as severe heart compressions, deformations, pericardial edema, and blood flow slowdown; these were the same as the phenotypes of the TU *dhfr* KI strain (Figure 6).

### Pathological Examination of Zebrafish

**Figure 7:**
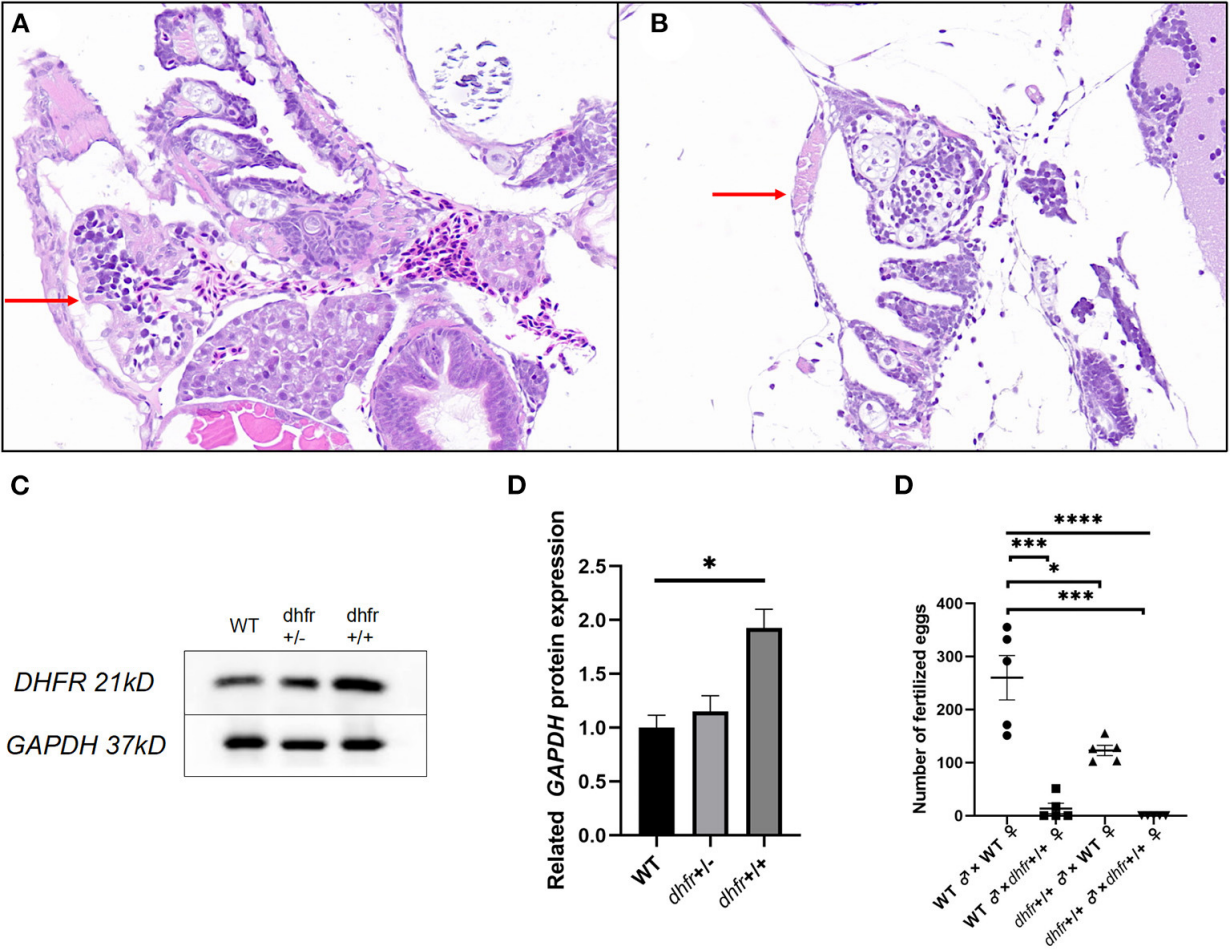
Results of pathology, western blot, and hybridization experiments. **(A)** TU zebrafish; **(B)** F1 dhfr heterozygous self-progeny (the red arrow points to the heart area). **(C,D)** WT, *dhfr* KI heterozygote, and *dhfr* KI homozygous DHFR protein expression analysis (GAPDH was used as an internal control). E: impaired fertility in zebrafish knocked in by *dhfr* (^*^P < 0.05, ^***^P < 0.001, ^****^P < 0.0001).

The H&E stained pathological section results showed that the individual hearts of KI the zebrafish, were relatively small and contained linear malformations, pericardial edema, and other defective phenotypes (Figures 7A,B).

### *Dhfr* Protein Expression in *dhfr* KI Strain Zebrafish

To detect the changes in zebrafish heart protein expressions after *in vitro dhfr* gene KI, and to determine whether KI was successful, western blot experiments were used to verify the expression of the *dhfr* gene in the heart. The expression of the *DHFR* protein in the hearts of homozygous zebrafish with *dhfr* KI was significantly higher (*p* = 0.024) than in those of WT zebrafish, with an average expression increase of 1.93 times. Heterozygous and WT zebrafish were found to be not statistically different, but the average expression increased by 1.15 times (Figures 7C,D).

### Fertility of *dhfr* KI Zebrafish

Compared with WT pairs, WT females/KI males and KI females/WT males produced lower numbers of eggs per mating, and KI pairs did not produce any fertilized eggs at all. There were statistically significant differences between the fertilized eggs laid by each group (*p* < 0.0001). The number of crosses indicated that there were statistically significant differences between KI, KI male/WT female, KI female/WT male, and WT pairs (*p* = 0.0004, *p* = 0.0127, and *p* = 0.0003, respectively). Whenever KI females were involved, the number of fertilized eggs was significantly lower (Figure 7E).

## Discussion

At present, it is believed that CHDs are complex diseases with abnormal cardiovascular developments caused by multiple factors (such as genetics and the environment), among which genetic factors are thought to play a major role. CHDs caused by genetic factors are generally divided into three subtypes: Mendelian single gene-diseases (such as splicing sites or exon base changes; the size is generally one base) such as *GATA4, NKX2-5, TBX1, JAG1, FOG2, ALK2, GDF1, TDGF1, FOXH1*, and *ZIC3*; and other gene mutations that can cause conical stem deformities (13–15). The second subtype contains chromosomal diseases (generally more than 3 million bases per 3 Mb in size), such as Downs syndrome (16). The last type constitutes genomic diseases (~10–3 million bases per 1 kb−3 Mb in size) (17). Genome rearrangement leads to the duplication or deletion of gene CNVs (18). Studies of genetically engineered mouse models have shown that more than 1,700 genes are involved in heart development, but to date only a few gene mutations have been confirmed to be related to human cardiovascular diseases (19). In recent years, many studies have shown that CNVs play an important role in the genetic pathogeneses of various congenital malformations.

PA is an extreme type of disease in the right heart system disease spectrum of CHDs; it is a conical malformation that occurs at an incidence of ~21.4 cases per 100,000 people (20). PA includes PA with a ventricular septal defect, PA with an intact ventricular septum, and PA with other complex heart malformations. Among them, PA with a ventricular septal defect is the most common type. PA, tetralogy of Fallot, double outlet of the right ventricle, transposition of the great arteries, common trunk, interruption of the aortic arch, and congenitally corrected transposition of the great arteries are all conical trunk malformations. Such malformations are caused by stagnation or interference in the development of the bulbar segment (conus trunk segment) in the embryonic stage (21, 22).

There are various causes of conotruncal defects including PA, but distinct genetic elements responsible for the pathogenesis of conotruncal defects have not yet been elucidated. However, patients with some syndrome, provide a reference to study the etiology of conotruncal defects. For example, ~15% of patients with TOF also suffer from 22q11.2 deletion syndrome (23). The most common congenital heart diseases for 22q11.2 deletion syndrome is conotruncal defects, such as Tetralogy of Fallot (TOF, 20–45%) and PA (10–25%) (24). A recent study found that 15.9% of 251 patients with 22q11.2 deletion syndrome had TOF deletions. This was almost equivalent to the proportion of patients with TOF, having 22q11.2 deletion reported in most studies. Therefore, it is speculated that certain genes are involved in pyramidal stem deformity in this region. Among all the ideal candidates, the primary pathogenic gene is *TBX1* (25). Studies have demonstrated that *TBX1* expression is essential in pharyngeal mesoderm for the pharyngeal segmentation and the normal outflow tract development. TBX1 promotes increased proliferation and delayed differentiation of the second heart field cells thereby affecting the embryonic development (26). Moreover, some studies have reported that knockout and overexpression of *Tbx1* in mouse results in the phenotypes of 22q11.2 deletion syndrome (27, 28). The effect of *TBX1* is similar to that of the *DHFR* gene, and alterations in *DHFR* dosage may cause the phenotype of PA. Therefore, based on these current findings it could be inferred that the 22q11.2 deletion syndrome is the most common genetic cause of PA. In addition, Down syndrome and Vacterl syndrome have also been shown to be associated with PA (29). Furthermore, in previous reports, we and several other groups have elucidated diverse factors contributing to PA (30).

In addition to the CNV mechanism, there are other types of genetic mutations that may cause PA. For example, mutations in *NKX2-5, GATA4* and *TBX5* genes can cause conotruncal defects and are intricately associated with PA (31–33). In addition to these common genes, other important regulatory genes, such as *NOTCH1* have recently been reported to be closely related to PA as revealed in Genome wide association study analysis of TOF whole exome sequencing data (23). The association of NOTCH1 with a series of heart defects corroborates its reported role in heart development. Thus, active NOTCH1 expression is observed in the trabecular endocardium. Moreover, global and endothelial-specific knockout of *Notch1* in mice results in abnormal trabecular ventricles as well as abnormal patterns of cardiomyocytes (34). With regard to TOF, NOTCH1 plays a crucial role in the tissues of the outflow tract, which requires cellular specification of the neural crest and secondary heart field (35). In addition, NOTCH1 is necessary for endocardial-epithelial-mesenchymal transition, a process that is essential for heart valve formation (36). These findings are of great significance and provide valuable insights which could be useful for further research on conical deformity.

With improvements in genetic testing and editing technologies, more and more candidate pathogenic genes and development mechanisms of genetic diseases have been clarified. The latest gene-editing technology, CRISPR/Cas9, has been widely used in studies of various gene knockout biological models such as mice, zebrafish, and chicken (37). Zebrafish have many of the same characteristics as other vertebrates regarding their anatomy and physiology, and have the advantages of being small individuals that reproduce easily, develop quickly, and produce transparent embryos. As zebrafish have great advantages for the effective study of the genetic development of vertebrate hearts, they have become an ideal model animal for studying genes related to heart development (38).

A previous study used array data analysis to detect that the 5q14.1 micro-repeat segment contained the *DHFR* gene. This suggested that a CNV of the *DHFR* gene may be a candidate pathogenic mechanism for PA. Subsequently, the present study used CRISPR/Cas9 technology to establish a zebrafish strain of *dhfr* KI, so that *dhfr* was overexpressed in zebrafish. This permitted the investigation of whether *dhfr* CNVs play a role in heart development. Light microscopy revealed that F3 generation KI zebrafish individuals had severe heart compressions, deformations, pericardial edema, and blood flow slowdown at 6 dpf, indicating that the *dhfr* gene plays an important regulatory role in the development of the cardiovascular system. Western blot analysis confirmed that the expression of *DHFR* protein in the hearts of zebrafish homozygous and heterozygous for *dhfr* KI increased compared with that of WT zebrafish, and the expression of *DHFR* protein in homozygous and heterozygous *dhfr* KI also increased. HE staining of adult zebrafish heart sections revealed that the hearts of KI individuals had defective phenotypes such as heart reductions. The shrinkage of the heart has a huge impact on cardiac hemodynamics. Observations also revealed that *dhfr* gene KI may cause abnormal gonadal function, especially in the ovaries. However, this needs further study. Therefore, the present study of zebrafish gene KI model organisms suggested that the abnormality of the *DHFR* gene affects the normal development of the heart, leading to abnormal or defective heart development.

The successful establishment of a *dhfr* KI zebrafish strain could permit the further construction of PA zebrafish model organisms, and be used to verify PA's occurrence and development mechanism, and could allow for the exploration of a new model organism for studying the pathogenic mechanisms of CHDs. As zebrafish embryos and juveniles are transparent, the green fluorescent protein gene can be transfected into zebrafish embryos together with the target gene. Researchers can thus study the expressions and functions of target genes, thereby allowing the exploration and verification of pathogenic signaling pathways. This will lay the foundation for further exploration of the molecular mechanisms of CHDs and their regulatory signal pathways.

The CNV of *DHFR* the gene studied in this article may cause the molecular pathogenesis of PA. To date there have been no reports on the role of *DHFR* CNV in heart development. The *DHFR* gene is located at 5q14.1 of chromosome 5, and the encoded protein is named dihydrofolate reductase. Dihydrofolate reductase has a total of 187 amino acids and is highly conserved. This protein is mainly expressed in mitochondria and converts dihydrofolate into tetrahydrofolate (39). There have been a large number of reports in the literature that *DHFR* gene mutations mainly cause resistance to antifolate drugs. Sun et al. found that the overexpression of the *dhfr* gene can partially improve the abnormal development of the heart and blood vessels of zebrafish embryos induced by ethanol. This may have been achieved by the up-regulation of decreases in the expressions of *nkx2.5, tbx1*, and *flk-1*, caused by ethanol (40). The regulation mechanisms of the *DHFR* gene for each exon and dihydrofolate reductase have not yet been fully elucidated. However, here *dhfr* KI zebrafish showed abnormalities such as cardiac edema and cardiac blood flow slowdown.

Our research has some limitations. First, we only constructed an animal model of zebrafish, and we have not further studied its mechanism. This aspect is also the direction of our future research. We hope to build cell models and conduct research on related mechanisms and pathways. Secondly, about the study of pathological results and sexual function, the amount of data is relatively small because pathological sections of zebrafish juveniles are difficult to cut to the heart, and the number of homozygotes *in vivo* is relatively small. At the same time, there are fewer homozygous adult fish, so there are fewer repetitions of the western blot results. We will continue to study the changes in the expression of their protein levels in the future. In the future, we will further supplement the data and continue to conduct in-depth research. At the same time, we hope that this copy number variant can be found in other patients in the future to further prove the function and pathogenicity of the alteration. Therefore, we will collect more relevant patients and perform CNV.

This experiment identified important genes of *dhfr* KI strain zebrafish related to heart development. Based on the results of previous studies, *DHFR* may be an important regulatory gene for heart development, and so could play an important role in the pathogenesis of CHD. The CNV of this gene may contribute to the pathogenic mechanism of CHD. Here, hybridization experiments revealed that *dhfr* CNV has an effect on the early heart development of zebrafish. One possible mechanism is that the *dhfr* CNV changes the expression of dihydrofolate reductase. This leads to abnormal cell folate metabolism, which then leads to abnormal one-carbon metabolism, nitric oxide, folate cycle, and cell cycle signaling pathways. This in turn, results in abnormal heart development and CHD. To further verify this speculation, efforts should be made in the future to construct a model mammalian organism for KI mice and observe the overall development of their hearts. These model organisms could be used to study obstacles that may be caused by one-carbon metabolism, nitric oxide, folate cycle, cell cycle, and other signaling pathways.

## Data Availability Statement

The datasets presented in this study can be found in online repositories. The names of the repository/repositories and accession number(s) can be found in the article/Supplementary Material.

## Ethics Statement

The animal study was reviewed and approved by the Institutional Animal Care and Use Committee (IACUC), the Second Xiangya Hospital Central South University, China.

## Author Contributions

KG, ZT, YY, YD, KC, and LX contributed to conception and design of the study. KG and TX completed the experiment, performed the statistical analysis, and wrote the first draft of the manuscript. KY, TX, YL, and HG wrote sections of the manuscript. All authors contributed to manuscript revision, read, and approved the submitted version.

## Funding

This study was supported by the National Science Foundation for Young Scientists of China (8150020951), the Natural Science Foundation for Young Scientists of Hunan Province (2016JJ4099), and the National Natural Science Foundation of China (31970504 and 31772548).

## Conflict of Interest

The authors declare that the research was conducted in the absence of any commercial or financial relationships that could be construed as a potential conflict of interest.

## Publisher's Note

All claims expressed in this article are solely those of the authors and do not necessarily represent those of their affiliated organizations, or those of the publisher, the editors and the reviewers. Any product that may be evaluated in this article, or claim that may be made by its manufacturer, is not guaranteed or endorsed by the publisher.

## Supplementary Material

Supplementary Figure 1 Ethical review form.

Supplementary Figure 2 Embryo count.

Supplementary Figure 3 Wb.
