# Supplementary figures and images for: Establishment of a Dihydrofolate Reductase Gene Knock-In Zebrafish Strain to Aid Preliminary Analysis of Congenital Heart Disease Mechanisms
Source: Front Cardiovasc Med. 2021 Dec 15;8:763851. doi: 10.3389/fcvm.2021.763851 (PMC8714833; doi:10.3389/fcvm.2021.763851)

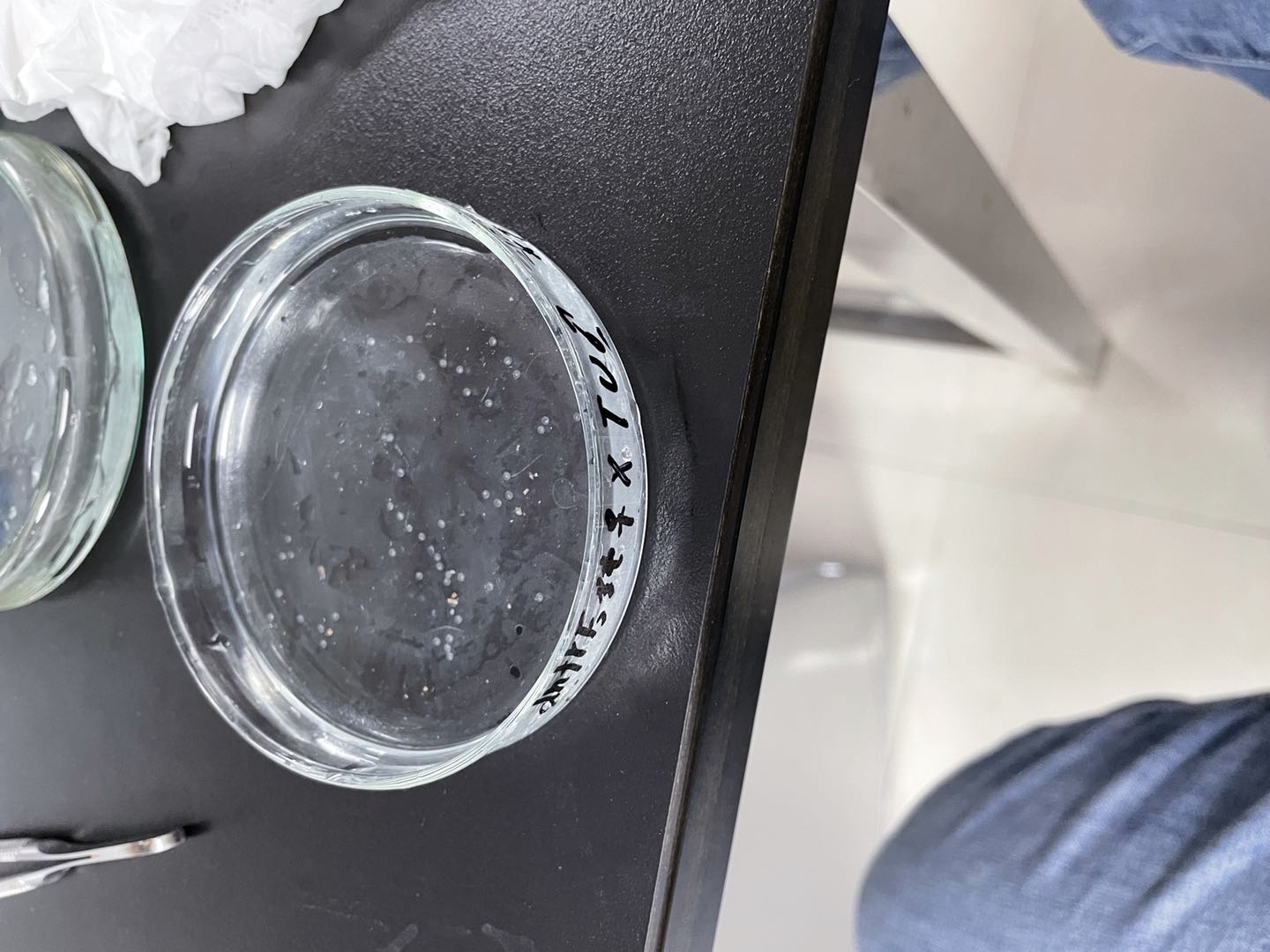

Supplement: Supplementary Figure 1 — Ethical review form. [file Data_Sheet_1.ZIP › Embryo count/dhfr female 51.jpg]

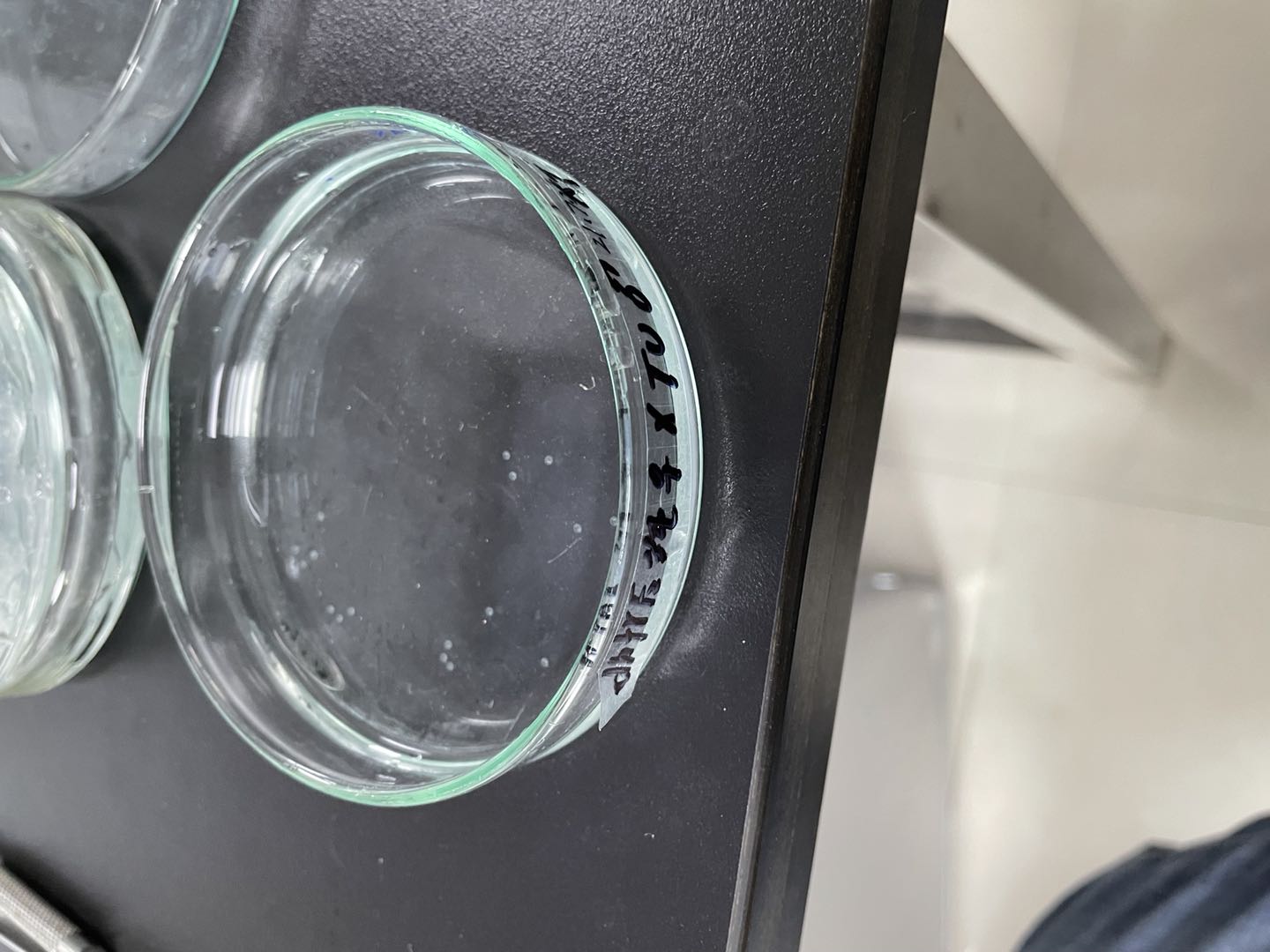

Supplement: Supplementary Figure 1 — Ethical review form. [file Data_Sheet_1.ZIP › Embryo count/dhfr female17.jpg]

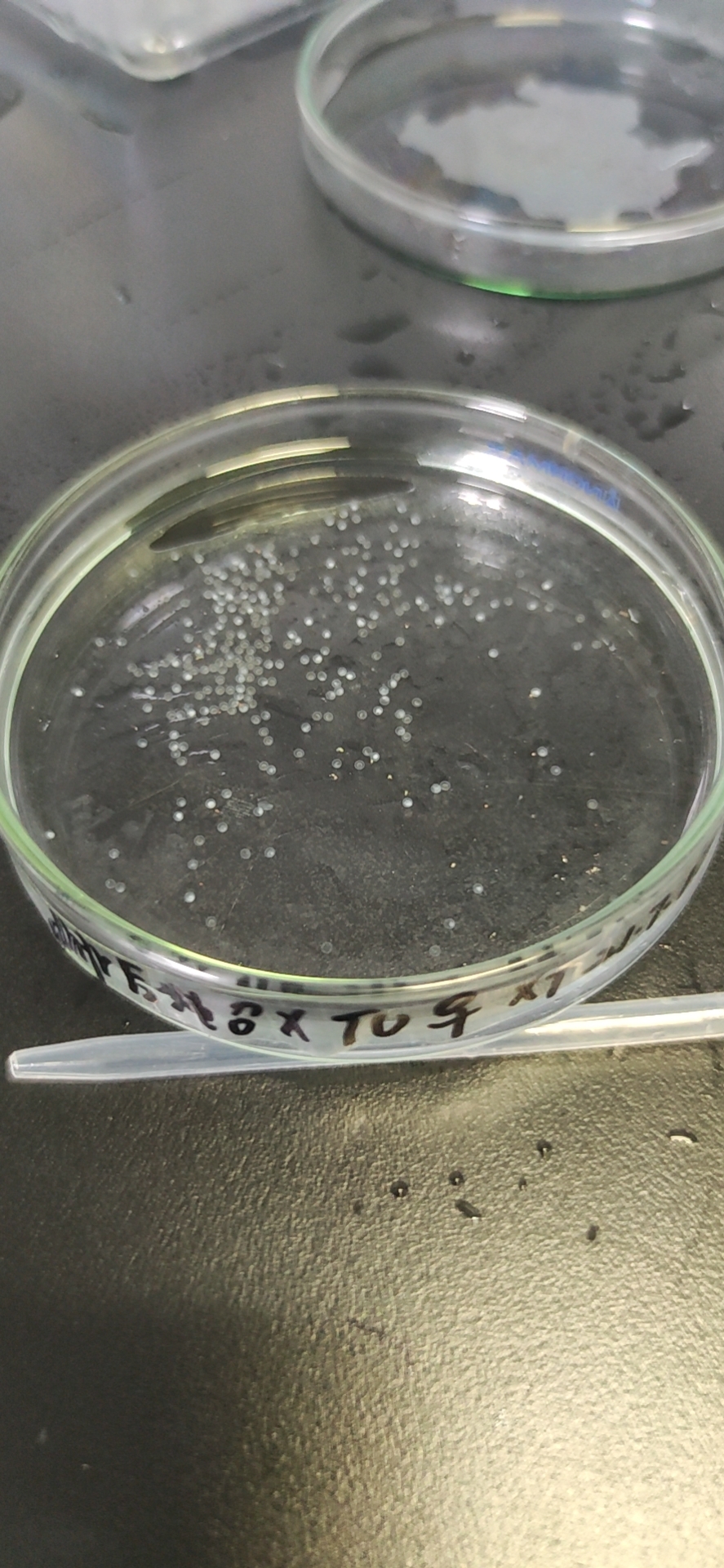

Supplement: Supplementary Figure 1 — Ethical review form. [file Data_Sheet_1.ZIP › Embryo count/dhfr male 155.jpg]

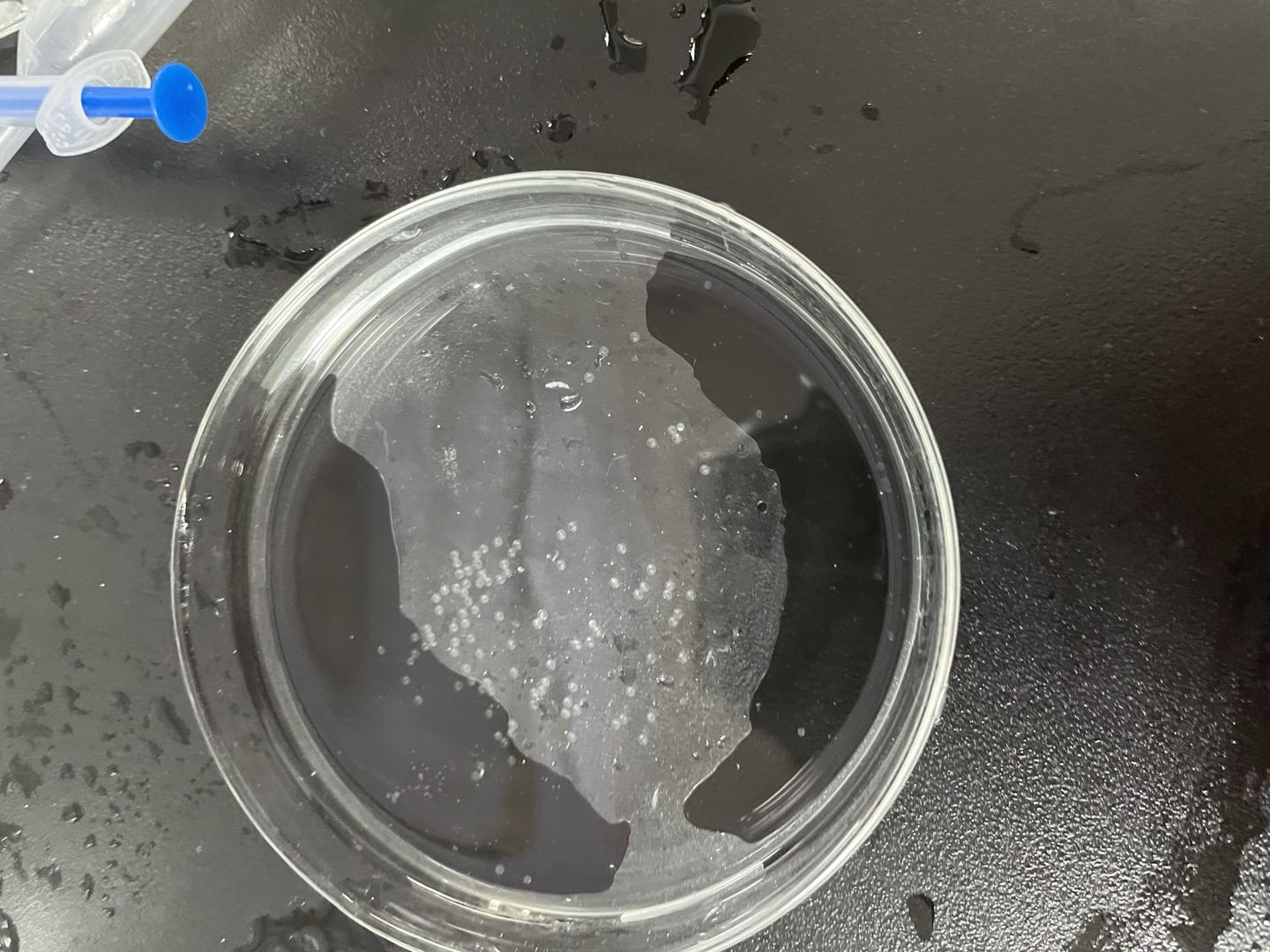

Supplement: Supplementary Figure 1 — Ethical review form. [file Data_Sheet_1.ZIP › Embryo count/dhfr male102.jpg]

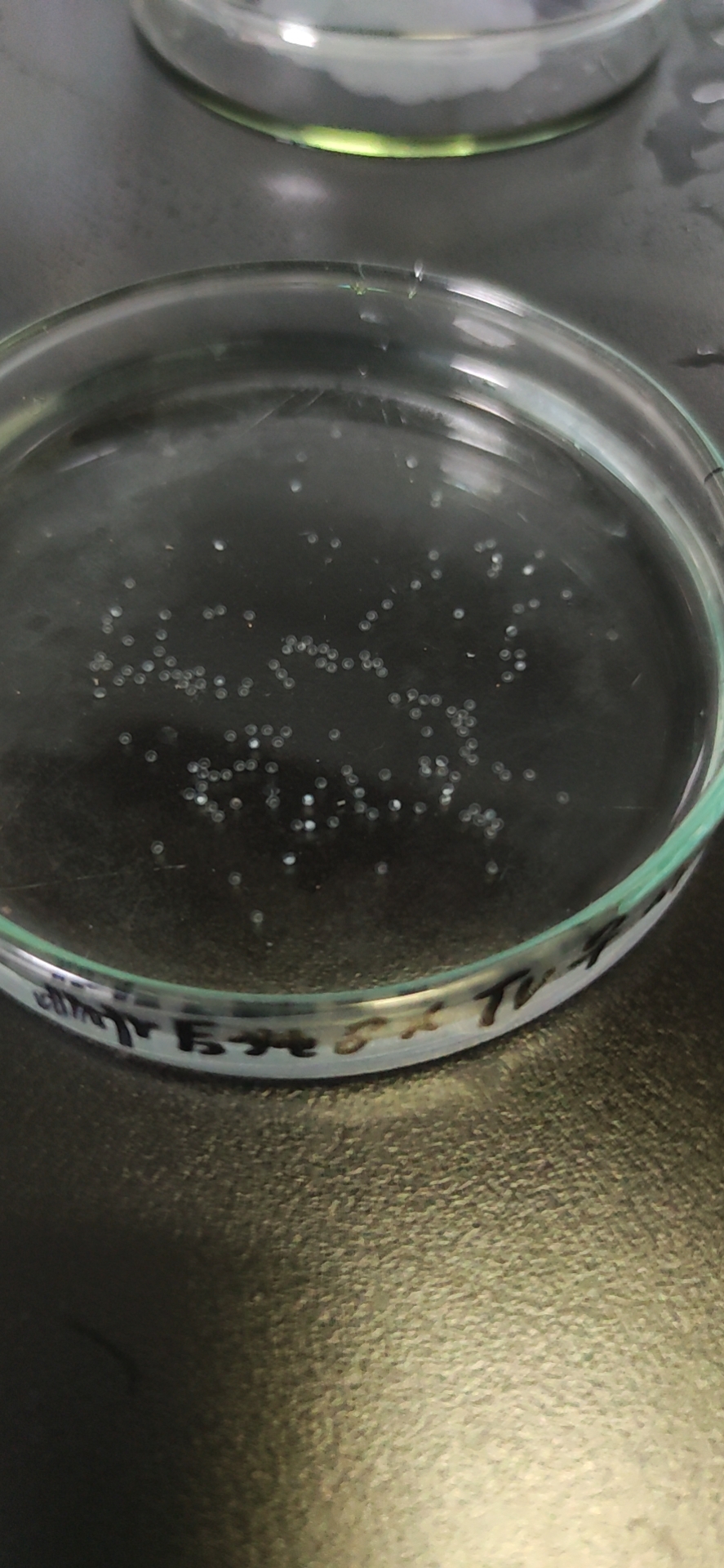

Supplement: Supplementary Figure 1 — Ethical review form. [file Data_Sheet_1.ZIP › Embryo count/dhfr male103.jpg]

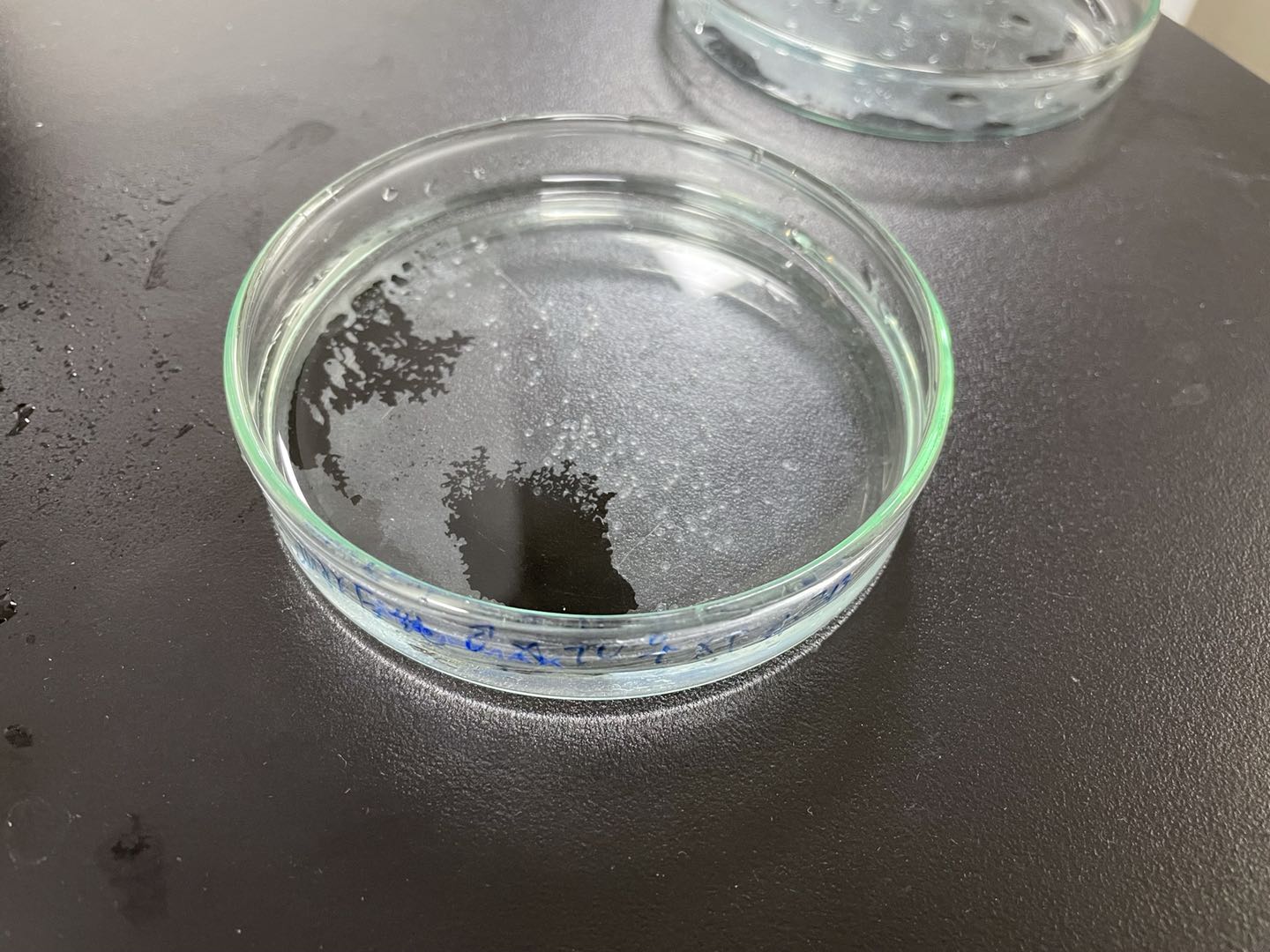

Supplement: Supplementary Figure 1 — Ethical review form. [file Data_Sheet_1.ZIP › Embryo count/dhfr male126.jpg]

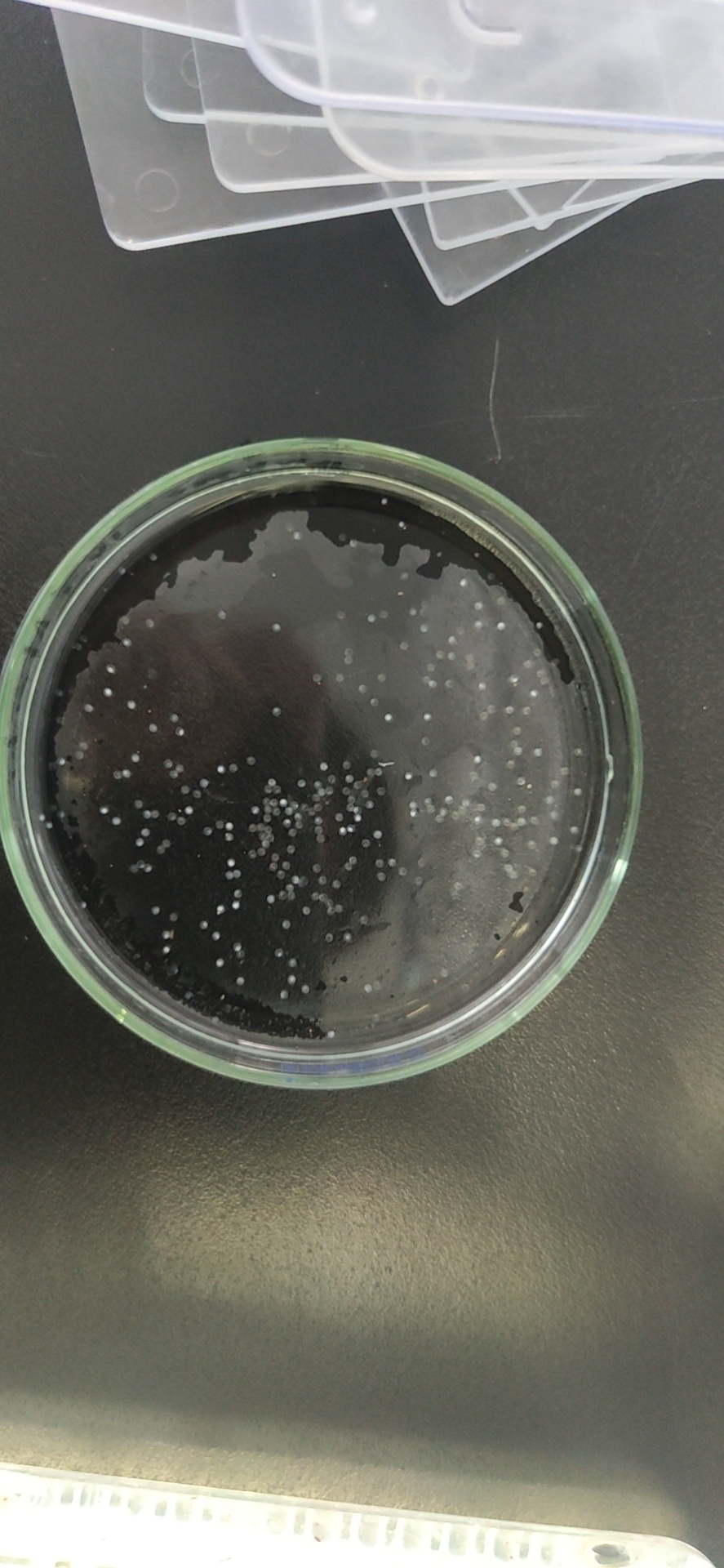

Supplement: Supplementary Figure 1 — Ethical review form. [file Data_Sheet_1.ZIP › Embryo count/dhfr male128.jpg]

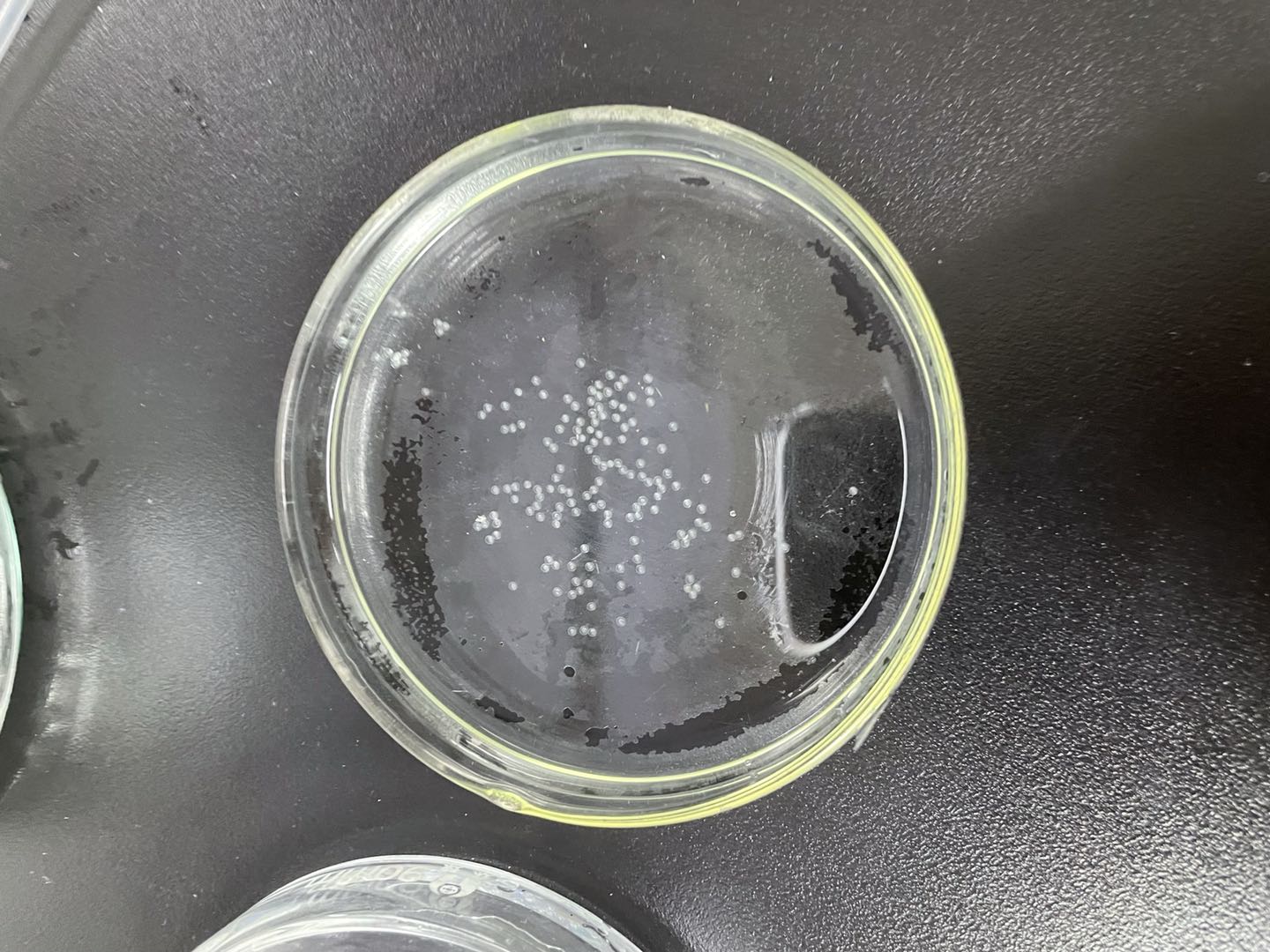

Supplement: Supplementary Figure 1 — Ethical review form. [file Data_Sheet_1.ZIP › Embryo count/tu 151.jpg]

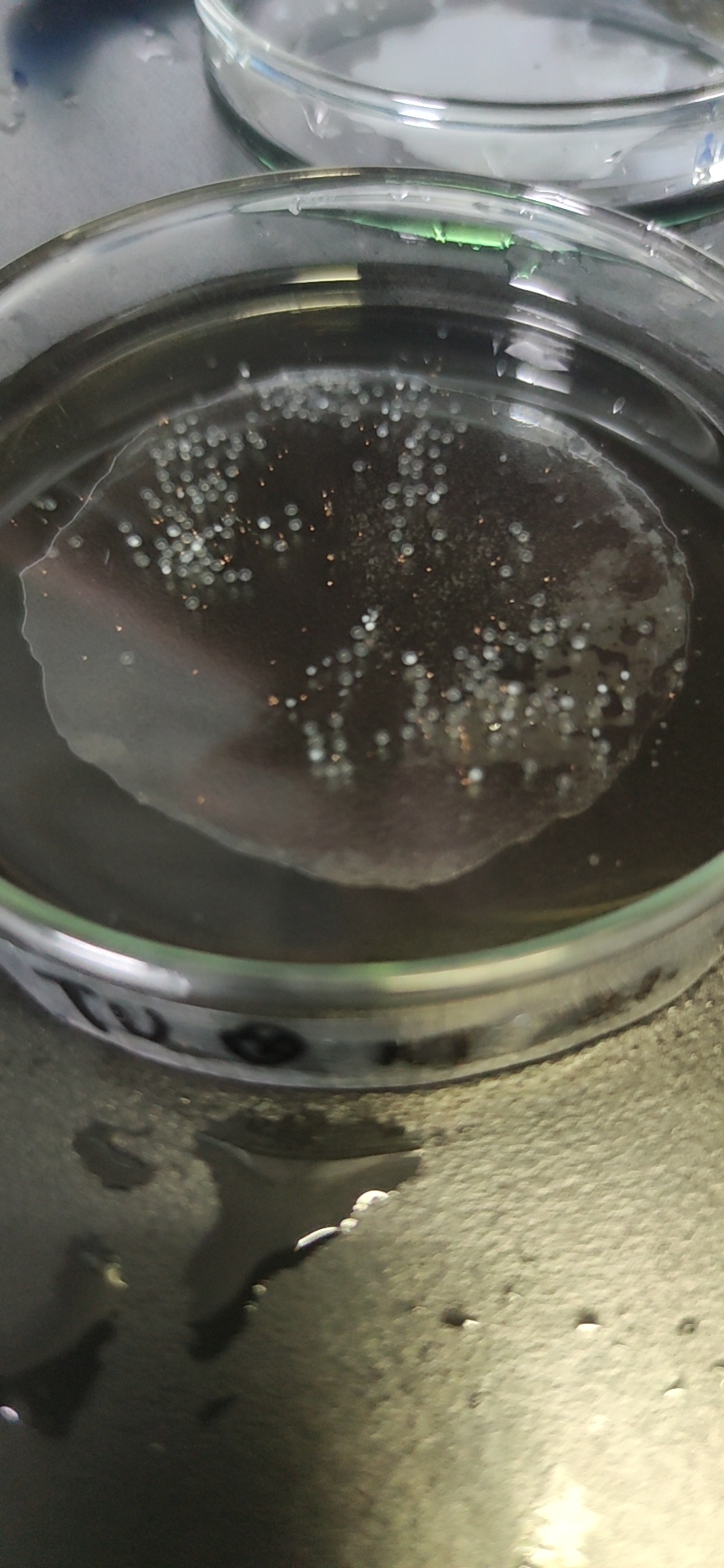

Supplement: Supplementary Figure 1 — Ethical review form. [file Data_Sheet_1.ZIP › Embryo count/tu 171.jpg]

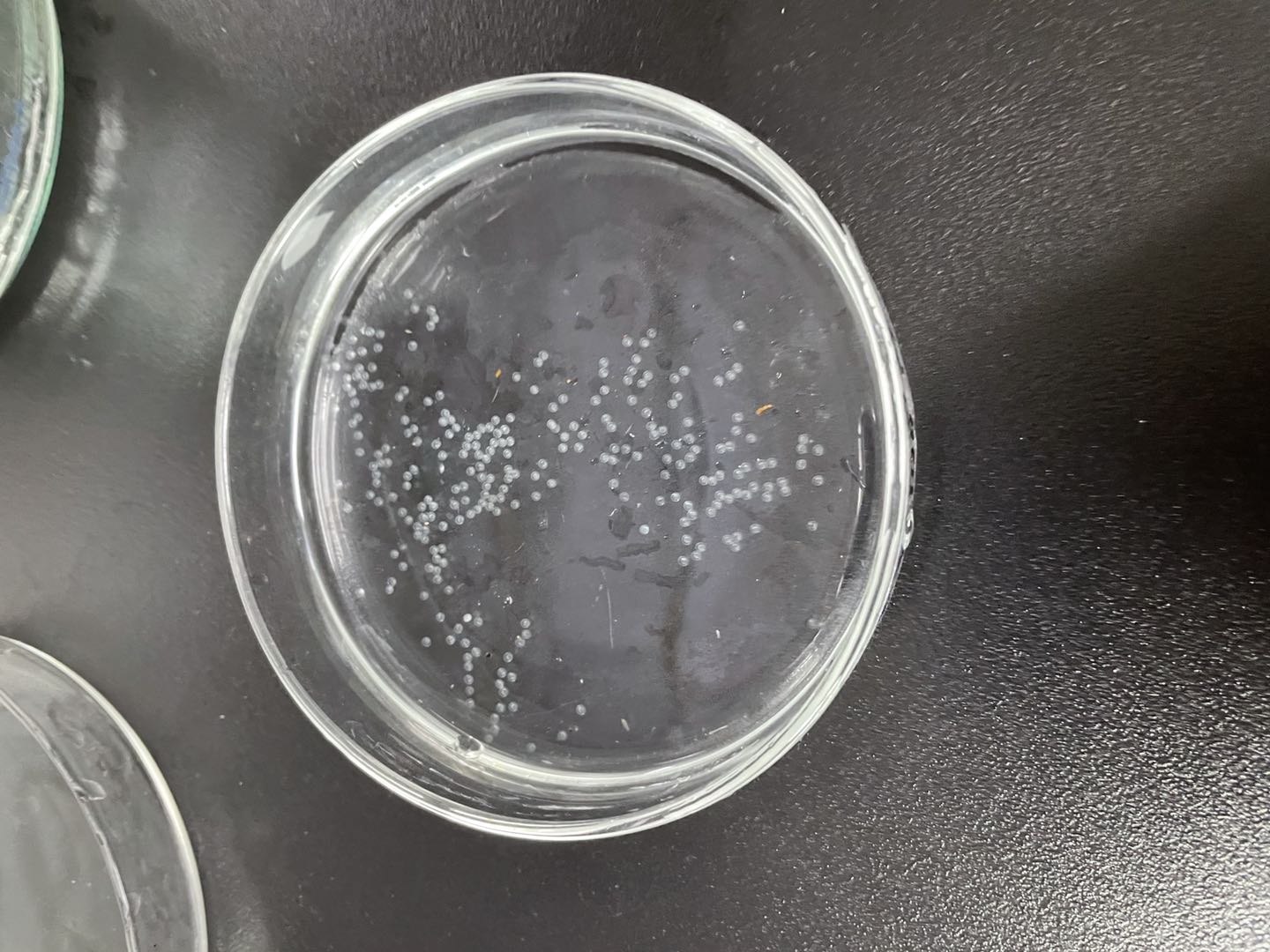

Supplement: Supplementary Figure 1 — Ethical review form. [file Data_Sheet_1.ZIP › Embryo count/tu 291.jpg]

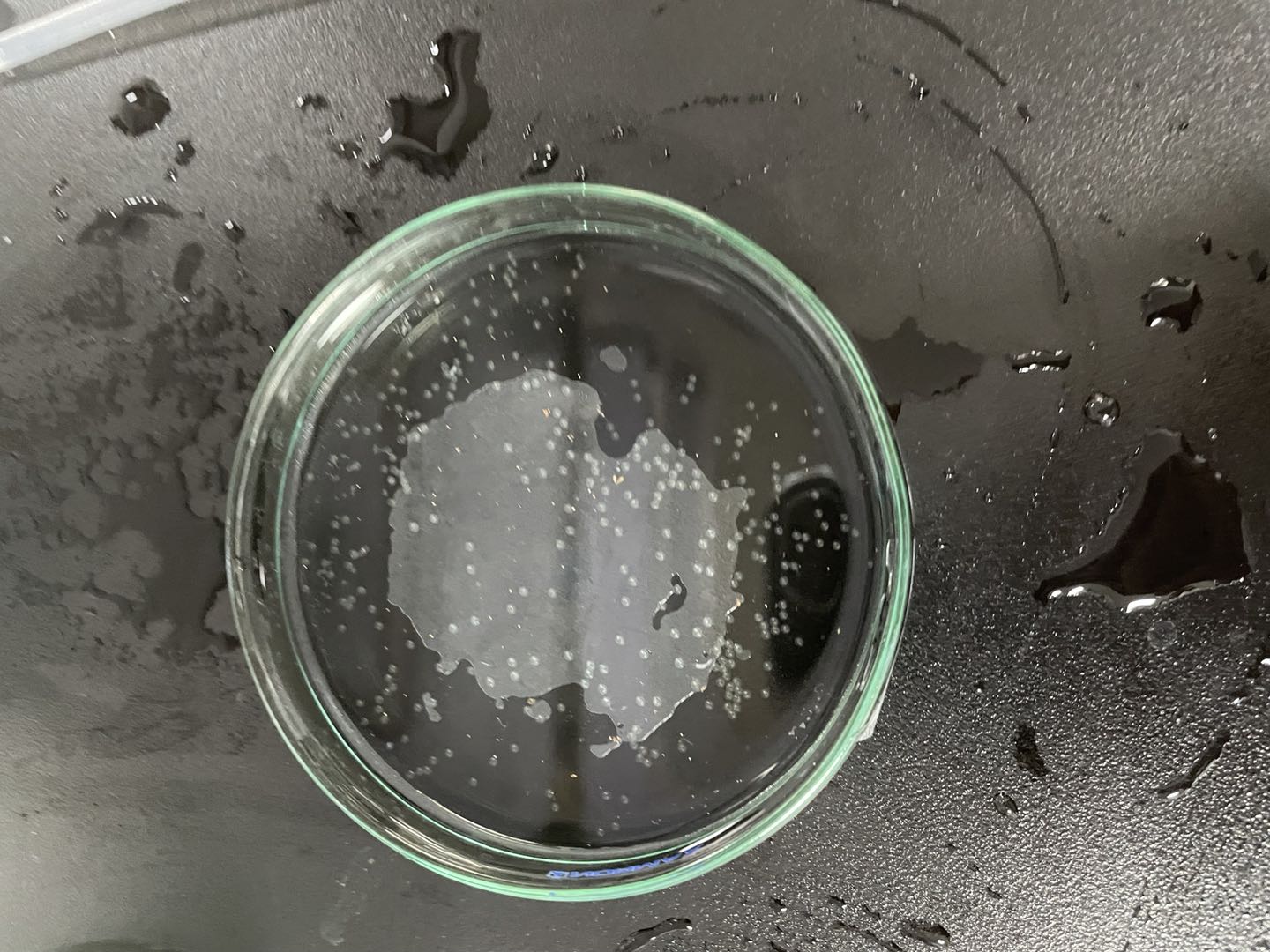

Supplement: Supplementary Figure 1 — Ethical review form. [file Data_Sheet_1.ZIP › Embryo count/tu 322.jpg]

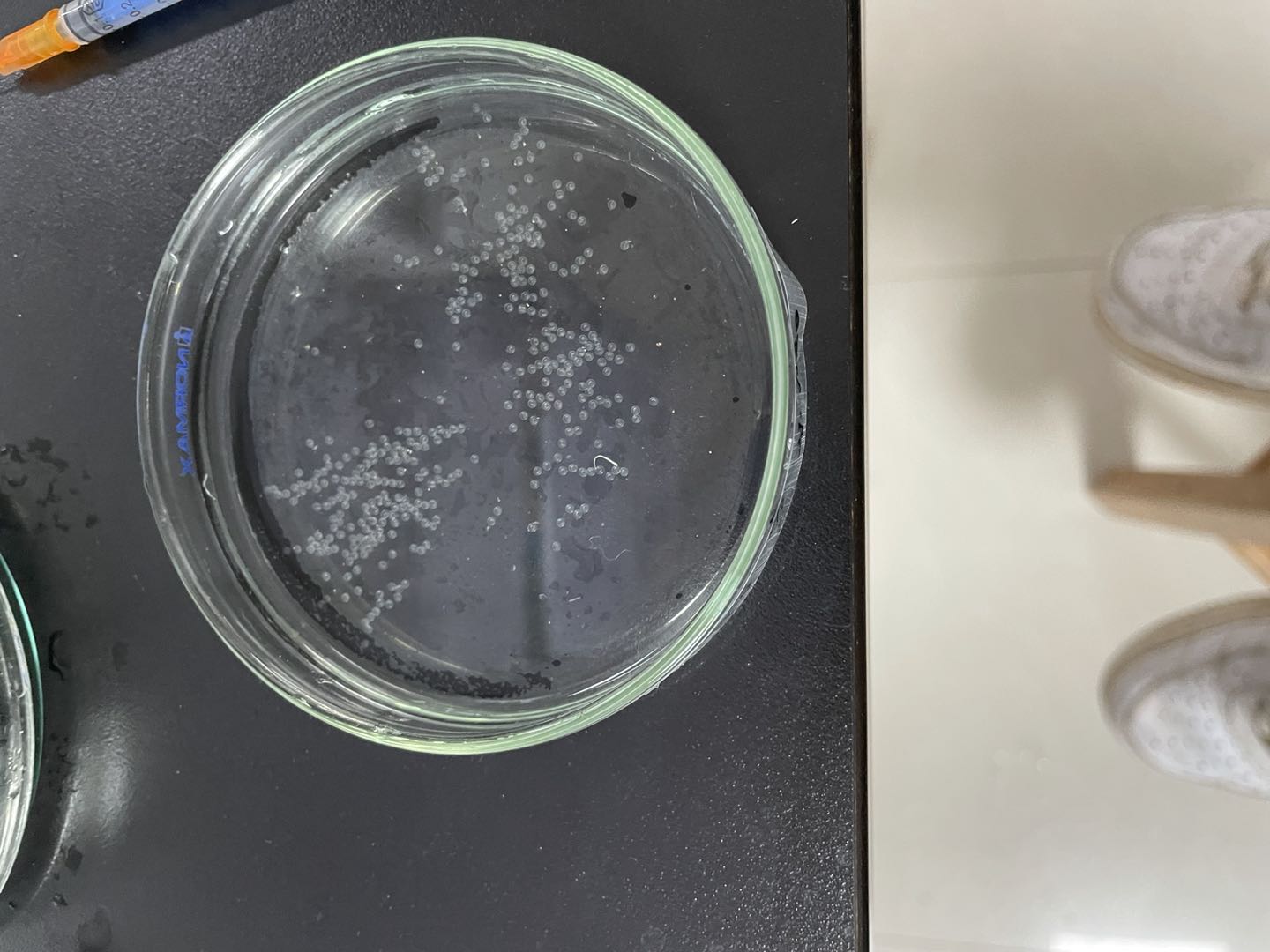

Supplement: Supplementary Figure 1 — Ethical review form. [file Data_Sheet_1.ZIP › Embryo count/tu 355.jpg]
